# Longitudinal Stress Hyperglycemia Trajectories and Severe Outcome in Patients Hospitalized for Viral Respiratory Infections: A 72-Hour Phenotyping Study

**DOI:** 10.3390/diagnostics16152310

**Published:** 2026-07-23

**Authors:** Ana Maria Mihai, Ovidiu Rosca, Florina Lucaciu, Alexandra Herlo, Talida Georgiana Cut, Matilda Radulescu, Delia Mira Berceanu-Vaduva, Ioana-Melinda Luput-Andrica, Radu Gheorghe Dan, Andra-Elena Saizu, Andreea-Cristina Floruncut, Adelina-Raluca Marinescu, Alexandra Sima

**Affiliations:** 1 Doctoral School, “Victor Babeș” University of Medicine and Pharmacy Timisoara, Eftimie Murgu Square 2, 300041 Timisoara, Romania; 2 Department XIII, Discipline of Infectious Diseases, “Victor Babeș” University of Medicine and Pharmacy Timisoara, Eftimie Murgu Square 2, 300041 Timisoara, Romania; 3 Victor Babes Clinical Hospital for Infectious Diseases and Pneumology of Timisoara, 300041 Timisoara, Romania; 4 Methodological and Infectious Diseases Research Center, Department of Infectious Diseases, “Victor Babeș” University of Medicine and Pharmacy Timisoara, Eftimie Murgu Square 2, 300041 Timisoara, Romania; 5 Department of Microbiology, Division of Microbiology, “Victor Babeș” University of Medicine and Pharmacy Timisoara, Eftimie Murgu Square 2, 300041 Timisoara, Romania; 6 Center for Hepato-Biliary-Pancreatic Surgery, “Victor Babeș” University of Medicine and Pharmacy Timisoara, 300041 Timisoara, Romania; 7 Department of Surgery I, “Victor Babeș” University of Medicine and Pharmacy Timisoara, 300041 Timisoara, Romania; 8 Second Department of Internal Medicine, “Victor Babeș” University of Medicine and Pharmacy Timisoara, 300041 Timisoara, Romania; 9 Department of Diabetes, Nutrition and Metabolic Diseases Clinic, “Pius Brînzeu” Emergency Clinical County University Hospital, 300723 Timisoara, Romania

**Keywords:** stress hyperglycemia ratio, diabetes mellitus, viral respiratory infections, prognostic biomarkers, metabolic resilience, respiratory failure, clinical triage

## Abstract

**Background/Objectives:** Patients with diabetes face increased risks during viral respiratory infections. While chronic glycemic control (HbA1c) is a known risk factor, it may fail to capture acute metabolic failure. This study aimed to evaluate the stress hyperglycemia ratio (SHR) at two time points (admission and 72 h) as a prognostic marker. **Methods:** We conducted a longitudinal analysis of 430 patients (211 with diabetes and 219 without diabetes) hospitalized with viral respiratory infections. The SHR was calculated at admission (SHR_z1) and at 72 h (SHR_z3). The primary outcome was severe clinical deterioration (defined as oxygen requirement >2–HFNO/OTI or mortality). Multivariate logistic regression and ROC curve analysis were used to determine independent predictors and clinical cut-off points. **Results:** In the global multivariable model (*N* = 430), continuous SHR_z3 emerged as an independent predictor of severe outcome (OR = 1.86, 95% CI 1.09–3.20, *p* = 0.0240) alongside chronic glycemic control (HbA1c: OR = 1.41, *p* < 0.0001) and age (OR = 1.05, *p* = 0.0001). After stratification, the independent SHR_z3 effect attenuated below statistical significance in both the non-diabetic (*p* = 0.6168) and diabetic (*p* = 0.1954) sub-cohorts, while HbA1c (*p* = 0.0404) and age (*p* = 0.0036) remained independent predictors within the diabetic subgroup. An exploratory longitudinal phenotype model classifying patients into Stable Normal, Resolvers, Persistent, and Late Spikers showed the highest in-sample discrimination of all models tested (AUC = 0.797, 95% CI 0.756–0.834). Within this model, the Persistent phenotype (SHR ≥ 1.1 sustained at admission and at 72 h) showed a non-significant trend toward higher adjusted odds of severe outcome compared with Stable Normal patients (OR = 1.81, 95% CI 0.91–3.60, *p* = 0.088) and was associated with a significantly longer hospital stay (Kruskal–Wallis *p* = 0.004 across phenotypes). **Conclusions:** Persistent stress hyperglycemia at 72 h is an independent predictor of severe outcome in the unstratified cohort after adjustment for chronic glycemic control, age, and other covariates. Within metabolic strata, the SHR_z3 signal attenuates, and long-standing suboptimal glycemic control (HbA1c) and age dominate, particularly among diabetic patients. Longitudinal SHR trajectories may therefore provide complementary, hypothesis-generating prognostic information when combined with chronic baseline markers, although these findings require external validation in independent cohorts before clinical use.

## 1. Introduction

The metabolic response of patients with diabetes mellitus (DM) during acute viral respiratory infections represents a critical determinant of clinical outcomes [1,2]. Patients with DM consistently exhibit heightened rates of multi-organ complications, prolonged intensive care utilization, and increased mortality during respiratory viral surges [3,4,5,6]. Traditional risk stratification has long relied on Glycated Hemoglobin (HbA1c) as a primary metric; however, while HbA1c effectively captures long-term chronic glycemic control, it is inherently limited by its static nature. It may not fully capture the body’s acute homeostatic failure or the immediate metabolic shock triggered by a severe viral infection [3,4,7].

The physiological response to severe viral infections is thought to involve a pronounced neuroendocrine cascade. Pro-inflammatory cytokines, including interleukin-6 (IL-6) and tumor necrosis factor-alpha (TNF-α), drive the release of counter-regulatory hormones such as cortisol and catecholamines [8]. This systemic inflammatory environment is proposed to impair pancreatic beta-cell function and to exacerbate peripheral insulin resistance, manifesting clinically as stress hyperglycemia (SH) [8]. Although transient SH functions as an evolutionary adaptive mechanism to supply energy to immune cells, persistent or excessive hyperglycemia can exacerbate pulmonary microvascular damage and worsen systemic inflammation [1,7,9]. These proposed mechanisms are drawn from the prior literature and are presented as background only; inflammatory biomarkers (e.g., IL-6 and TNF-α) and counter-regulatory hormones were not examined in the present analysis.

Despite its prognostic potential, absolute admission glucose levels can be highly misleading. In diabetic cohorts, baseline glucose levels are already elevated, making it difficult for clinicians to distinguish between chronic poor control and a true, acute stress-induced spike. The stress hyperglycemia ratio (SHR) was developed to control for chronic background hyperglycemia by indexing acute glucose values against estimated average background glucose derived from HbA1c [10]. While admission SHR snapshots (SHR_z1) reflect the immediate physiological response to viral exposure, they may not accurately predict structural clinical decline. Instead, the persistence of an elevated SHR beyond the initial 72 h of hospitalization may represent a failure of the host’s metabolic recovery mechanisms, coinciding with peak clinical or respiratory deterioration [11,12].

Conceptually, an SHR of approximately 1.0 indicates that acute glucose matches the HbA1c-derived chronic baseline, values above 1.0 denote relative (stress) hyperglycemia exceeding the patient’s own baseline, and values below 1.0 denote relative hypoglycemia; because it is normalized to each patient’s chronic glycaemia, the SHR is intended to separate a true, acute stress response from pre-existing chronic hyperglycemia, which absolute admission glucose cannot do. It should be acknowledged, however, that this 72 h landmark is extrapolated from the adjacent literature rather than directly validated in mixed viral respiratory infections: Reference [11] derives from septic ICU cohorts, and Reference [12] concerns diabetes prevalence and severity in COVID-19, so a specific 72 h SHR landmark in a mixed viral respiratory setting remains a translational gap that the present study begins to address.

Currently, longitudinal data evaluating the prognostic value of a persistent stress phenotype (SHR at 72 h/SHR_z3) versus admission metrics remain sparse, particularly regarding potential differences in metabolic resilience between diabetic and non-diabetic populations. This study aims to evaluate whether the longitudinal tracking of the SHR at 72 h provides independent prognostic value for clinical severity in patients hospitalized with PCR-confirmed viral respiratory infections, thereby establishing exploratory thresholds to optimize clinical monitoring [13,14]. This analysis was exploratory and hypothesis-generating rather than confirmatory. Our a priori hypotheses were that persistent stress hyperglycemia at 72 h (SHR_z3) would be associated with severe clinical outcome independently of chronic glycemic control (HbA1c) and age and that the strength of this association would differ between diabetic and non-diabetic strata; given the number of subgroup and threshold analyses performed, the resulting *p*-values should be interpreted in this exploratory context.

Evaluating these metabolic dynamics is of particular relevance within the Eastern European clinical context. In Romania and similar healthcare settings, the delayed diagnosis or suboptimal long-term monitoring of diabetes may contribute to high baseline HbA1c levels among hospitalized patients [15]. This prospective longitudinal study, conducted at the Victor Babeș Clinical Hospital for Infectious Diseases and Pneumology in Timișoara, aims to evaluate the real-world prognostic utility of acute stress hyperglycemia tracking versus chronic metabolic baselines within this distinct, highly vulnerable patient cohort. To our knowledge, this is among the first studies to track the SHR longitudinally across a 72 h landmark in a mixed, PCR-confirmed viral respiratory cohort and to test explicitly whether a persistent stress phenotype adds prognostic information beyond chronic HbA1c and age. The novelty lies less in confirming that persistent hyperglycemia accompanies severe disease than in quantifying how much independent signal the acute 72 h trajectory retains once chronic glycaemia is accounted for and in showing that this residual signal is concentrated in a definable longitudinal phenotype rather than in any single admission value.

## 2. Materials and Methods

### 2.1. Study Design and Patient Selection

**Figure 1:**
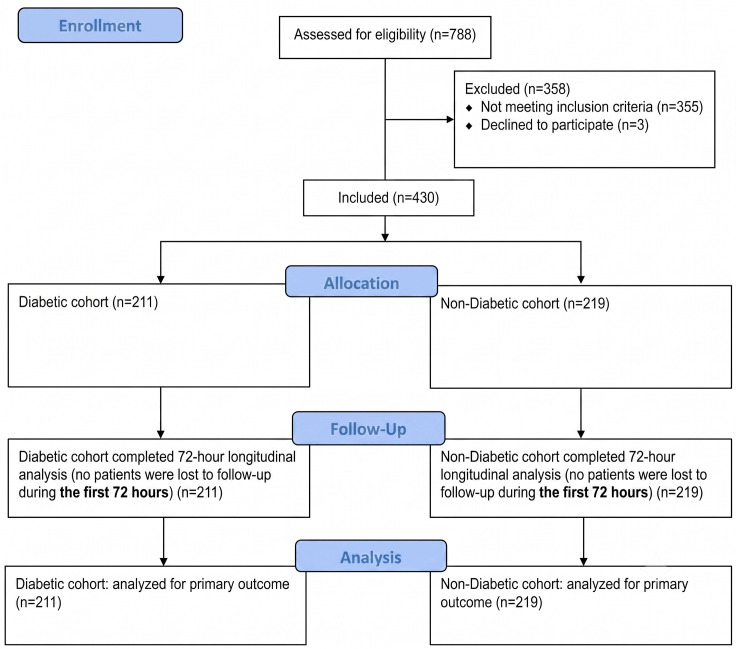
STROBE flow diagram of patient selection and study enrollment.

We conducted a prospective longitudinal observational study at the “Victor Babeș” Hospital for Infectious Diseases and Pulmonology in Timișoara, Romania. The study period spanned from 20 December 2024 to 1 April 2026, capturing multiple seasonal viral surges. The database was locked for analysis on 1 April 2026, after completion of in-hospital follow-up for all included patients (Figure 1). The study protocol was approved by the institutional Ethics Committee and internal review board (Approval No. 12066/12 December 2024). All participants provided written informed consent prior to enrollment.

Inclusion Criteria

• Adult patients (age ≥ 18 years) hospitalized with a confirmed viral respiratory infection.
• Viral identification was performed via multiplex PCR (BioFire^®^ FilmArray^®^ Respiratory Panel [16]) for a broad panel of respiratory pathogens, including SARS-CoV-2, Influenza A/B, RSV, and other seasonal respiratory viruses. Testing was performed on nasopharyngeal swab specimens collected at admission.

Exclusion Criteria

• Lack of a signed informed consent form.
• Absence of a positive multiplex PCR test to confirm the viral etiology.
• Any patient who died within the first 72 h of admission was excluded from the longitudinal analysis (no patient met this criterion within the first 72 h).
• Patients on continuous insulin infusions and patients on experimental regimens or any other experimental drugs.

Because SHR_z3 was measured at 72 h, prognostic analyses using SHR_z3 were performed as a 72 h landmark analysis. The primary objective of this analysis was to evaluate the systemic metabolic stress response (SHR) common to acute viral respiratory failure across the etiologies captured by the panel [8]. While a detailed breakdown of individual viral subspecies trajectories remains beyond the scope of this baseline analysis, multiplex PCR viral etiologies were aggregated into broad clinical–biological categories to serve as rigorous covariates within our multivariable adjustments [16,17].

### 2.2. Data Collection and Clinical Definitions

The study population was categorized into two cohorts: those with a pre-existing diagnosis of diabetes mellitus (*N* = 211) and non-diabetic controls (*N* = 219). Most patients in both cohorts presented with various comorbidities typical of an aging hospitalized population [18]. Commonly observed chronic comorbidities in both cohorts included arterial hypertension, ischemic heart disease and other cardiovascular diseases, COPD, chronic kidney disease, and in some patients, active or prior malignancy.

Treatment Standards

• Patients with Influenza or SARS-CoV-2 received standard-of-care protocol treatments according to national and international guidelines.
• Other viral infections were managed with symptomatic and supportive therapy.
• Enrolment spanned approximately 15 months and multiple seasonal surges; the same institutional diagnostic and management protocols were applied throughout, and standard-of-care recommendations for Influenza and SARS-CoV-2 in hospitalized adults did not change materially during this interval.

Oxygen Requirement Scale

The oxygen requirement grade refers to the maximum/peak respiratory support required by the patient during their stable course of hospitalization. Mortality was tracked independently of the maximum non-fatal oxygen support grade reached, capturing terminal events directly. Clinical severity was assessed using a standardized 5-point ordinal scale for respiratory support:

• Grade 0: No supplemental oxygen (room air).
• Grade 1: Supplemental oxygen via facial mask.
• Grade 2: Continuous Positive Airway Pressure (CPAP).
• Grade 3: High-Flow Nasal Oxygen.
• Grade 4: Orotracheal Intubation (OTI) and mechanical ventilation.

The primary composite endpoint was severe outcome defined as an oxygen requirement of >2 or all-cause in-hospital mortality.

### 2.3. Data Collection and Laboratory Analysis

Baseline demographics (age, sex, BMI, and abdominal circumference) and chronic glycemic status (HbA1c) were recorded at admission in the hospital [4,19]. The stress hyperglycemia ratio (SHR) was calculated at two critical time points to assess metabolic evolution [10,20,21]. Plasma glucose and glycated hemoglobin (HbA1c) were measured on an Abbott ARCHITECT c4000 clinical chemistry analyzer (Abbott Diagnostics, Abbott Park, IL, USA). Glucose was determined in venous plasma by the enzymatic hexokinase/glucose-6-phosphate dehydrogenase method with photometric detection. HbA1c was measured in EDTA whole blood using the automated ARCHITECT HbA1c assay (enzymatic):

1. SHR_z1: Calculated using glucose levels obtained at hospital admission.
2. SHR_z3: Calculated using glucose levels obtained 72 h post-admission; the 72 h glucose was a fasting measure.

The two primary formulas are utilized depending on the units used for glucose [20,21]. In the present study, plasma glucose was reported in mg/dL, and the corresponding formula (shown below) was applied uniformly to both the admission (SHR_z1) and 72 h (SHR_z3) values to ensure reproducibility:

1. For glucose in mg/dL:
  $$SHR=\frac{Admission\;Glucose\;(mg/dL)}{28.7\times HbA1c\left(\%\right)-46.7}$$
2. For glucose in mmol/L:
  $$SHR=\frac{Admission\;Glucose\;(mmol/L)}{1.59\times HbA1c\left(\%\right)-2.59}$$

### 2.4. Statistical Analysis

All statistical analyses were performed using MedCalc^®^ version 23.5.5 [22]. The reporting of this prospective cohort study adheres strictly to the Strengthening the Reporting of Observational Studies in Epidemiology (STROBE) statement guidelines. Continuous variables were expressed as medians with interquartile ranges (IQRs) and compared using the Mann–Whitney U test, while categorical variables were analyzed via the Chi-squared (χ^2^) test or Fisher’s exact test. Non-normality of the SHR_z1 and SHR_z3 distributions was confirmed by the D’Agostino–Pearson test (all *p* < 0.001), justifying the use of non-parametric methods for these variables. Prognostic accuracy was evaluated using Receiver Operating Characteristic (ROC) curve analysis to calculate the Area Under the Curve (AUC) for HbA1c, SHR_z1, and SHR_z3 [8]. Pair-wise comparisons of global AUC metrics were executed via the DeLong method. Optimal clinical cut-off thresholds for predicting the composite severe outcome were identified using the Youden index (J) across the total cohort and within isolated metabolic strata. To evaluate independent predictors of clinical severity, multivariable logistic regression models were constructed utilizing the Enter method.

To control for pathogen heterogeneity without over-fitting the model due to sparse data cells or quasi-complete separation, individual multiplex PCR viral etiologies were aggregated into four clinical–biological categories [23]: (1) Influenza and Parainfluenza viruses: Influenza A, AH3, AH1-2009 Influenza B, and Parainfluenza 1–4 (Reference Standard); (2) Coronaviruses: SARS-CoV-2, OC43, NL63 and HKU1; (3) Pneumoviruses: RSV and hMPV; and (4) other viral pathogens: Rhinovirus/Enterovirus and Adenovirus. To unmask the specific impacts of acute versus chronic glycemic indices, symmetrical multivariable models were executed across the total cohort (*N* = 430) and within isolated strata for non-diabetic (*n* = 219) and diabetic (*n* = 211) populations. Comparisons of continuous variables across more than two groups (e.g., length of stay across the four SHR phenotypes) were performed using the Kruskal–Wallis test, with Conover post hoc analysis for pair-wise comparisons.

## 3. Results

### 3.1. Baseline and Longitudinal Metabolic Characteristics

This study included 430 patients, divided into a diabetic cohort (*N* = 211) and a non-diabetic cohort (*N* = 219). Diabetic patients were significantly older and presented with higher metabolic markers (BMI and abdominal circumference) compared with non-diabetic individuals (Table 1). While initial stress responses (SHR_z1) were comparable between cohorts (*p* = 0.864), a significant metabolic divergence emerged by the 72 h mark. Non-diabetic patients demonstrated a trend toward metabolic resolution (median SHR_z3 of 0.88, IQR 0.81–1.00), whereas the diabetic cohort exhibited persistent stress hyperglycemia (median SHR_z3 of 1.07, IQR 0.76–1.32; *p* = 0.0002). This pattern suggests that while the initial viral insult triggers a comparable acute stress response across both cohorts, the persistence of this response beyond the first 72 h is a distinguishing feature of the diabetic population. Detailed breakdowns of categorical clinical milestones, including required oxygen support categories and in-hospital mortality rates across both cohorts are summarized (see Table 5 below). Serial inflammatory and coagulation markers (interleukin-6, C-reactive protein, procalcitonin, and D-dimer) were recorded at admission and on day 6 but are reserved for a separate, dedicated longitudinal analysis and are therefore not examined here.

**Figure 2:**
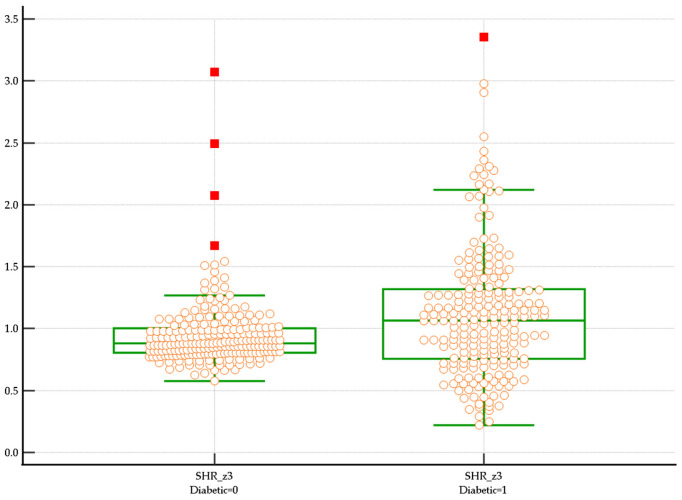
Cross-sectional distribution of the stress hyperglycemia ratio at 72 h (SHR_z3) stratified by diabetic status. The box plots with overlaid individual data points (*N* = 430) show that non-diabetic patients (Diabetic = 0; *n* = 219) cluster around metabolic resolution (median of 0.88, IQR 0.81–1.00), while diabetic patients (Diabetic = 1; *n* = 211) display a wider spread and persistently elevated SHR_z3 (median of 1.07, IQR 0.76–1.32; Mann–Whitney *p* = 0.0002), representing a sustained stress phenotype.

Chronic comorbidities were not systematically recorded in this analysis and were therefore not formally compared between the cohorts; the baseline characteristics that were captured (age, sex, BMI, abdominal circumference, and HbA1c) are summarized in Table 1. The distinctive cross-sectional variations in the SHR at 72 h are captured in Figure 2.

### 3.2. Independent Predictors of Severity

To evaluate the global independent prognostic value of acute stress hyperglycemia trajectories against established chronic markers, a multivariable logistic regression model was constructed for the entire cohort (*N* = 430) utilizing the consolidated four-group viral variable to eliminate sparse data cells. The model demonstrated acceptable overall calibration (Hosmer–Lemeshow χ^2^ = 13.26, df = 8, *p* = 0.1031) and achieved an Area Under the ROC Curve (AUC) of 0.771 (95% CI 0.729–0.810, *p* < 0.0001; Table 2).

**Figure 3:**
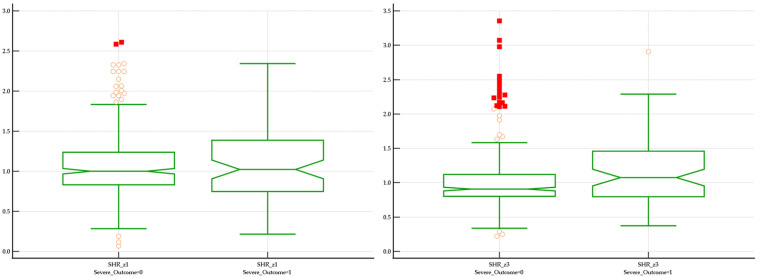
Median SHRs with 95% confidence intervals in patients with severe versus non-severe outcome at admission and at 72 h. At admission, SHR_z1 did not differ between outcome groups (median of 1.00 vs. 1.02; Mann–Whitney *p* = 0.9615). By the 72 h time point, a significant divergence emerged (SHR_z3 median of 0.91 in non-severe outcome group vs. 1.08 in severe outcome group; *p* = 0.0113), with severe outcomes characterized by persistent stress hyperglycemia, while non-severe patients demonstrated metabolic resolution.

In the fully adjusted global model, the continuous 72 h stress hyperglycemia ratio (SHR_z3) emerged as an independent predictor of severe clinical outcome (*p* = 0.0240, OR = 1.86, 95% CI 1.09–3.20), with each unit increase in SHR_z3 nearly doubling the odds of severe outcome after adjustment for chronic glycemic control, age, sex, BMI, abdominal circumference, and viral group. Chronic glycemic exposure (HbA1c: *p* < 0.0001, OR = 1.41, 95% CI 1.23–1.62) and advanced age (*p* = 0.0001, OR = 1.05, 95% CI 1.02–1.08) remained the strongest independent drivers. Biological sex, body mass index, abdominal circumference, and specific viral groupings did not emerge as independent drivers of severity within the global population step. The longitudinal deviations in median stress hyperglycemia ratios across clinical severity groups, alongside their respective 95% confidence intervals, are graphically tracked in Figure 3. These covariates were retained in every model a priori as pre-specified demographic and clinical confounders (age, sex, BMI, abdominal circumference, HbA1c, and viral group), to ensure symmetrical adjustment across the total cohort and both strata irrespective of their significance in any single model.

### 3.3. ROC Curve Analysis and Clinical Thresholds

When the multivariable model was fitted separately within metabolic strata (Table 3), the global independent SHR_z3 effect attenuated and no longer reached statistical significance in either the non-diabetic (*p* = 0.6168, OR = 1.64) or diabetic (*p* = 0.1954, OR = 1.51) sub-cohort. In the diabetic stratum (62 severe events), HbA1c (*p* = 0.0404, OR = 1.21) and age (*p* = 0.0036, OR = 1.05) remained independent predictors. In the non-diabetic stratum (only 12 severe events), no covariate reached statistical significance, consistent with limited statistical power. Overall calibration was preserved in both stratified models (Hosmer–Lemeshow *p* = 0.7410 and *p* = 0.1348, respectively). With only 12 severe events against multiple predictors, the events-per-variable ratio in the non-diabetic stratum is approximately 1.2, far below the conventional minimum of 10; the resulting estimates are unstable, as reflected in the very wide confidence intervals (e.g., OR 3.87, 95% CI 0.60–25.04), so the non-diabetic stratified model should be regarded as descriptive/exploratory only and not used for inference.

**Figure 4:**
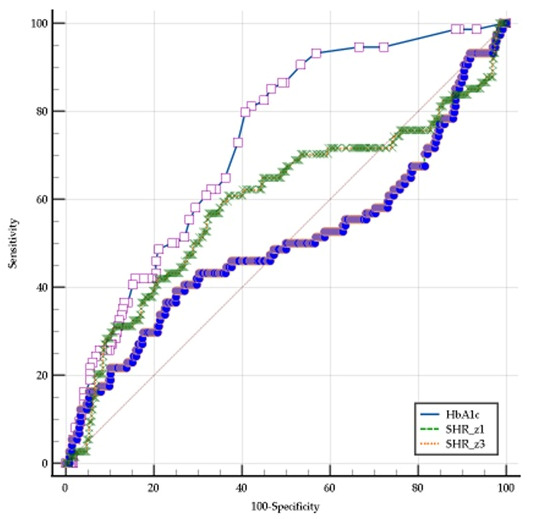
Comparison of Receiver Operating Characteristic (ROC) curves of HbA1c, admission SHR (SHR_z1), and 72 h SHR (SHR_z3) in prediction of severe outcome in the total cohort (*N* = 430). AUCs: HbA1c, 0.730 (95% CI 0.686–0.772); SHR_z1, 0.502 (0.453–0.550); SHR_z3, 0.594 (0.545–0.640). DeLong pair-wise comparisons reported in Table 4.

**Figure 5:**
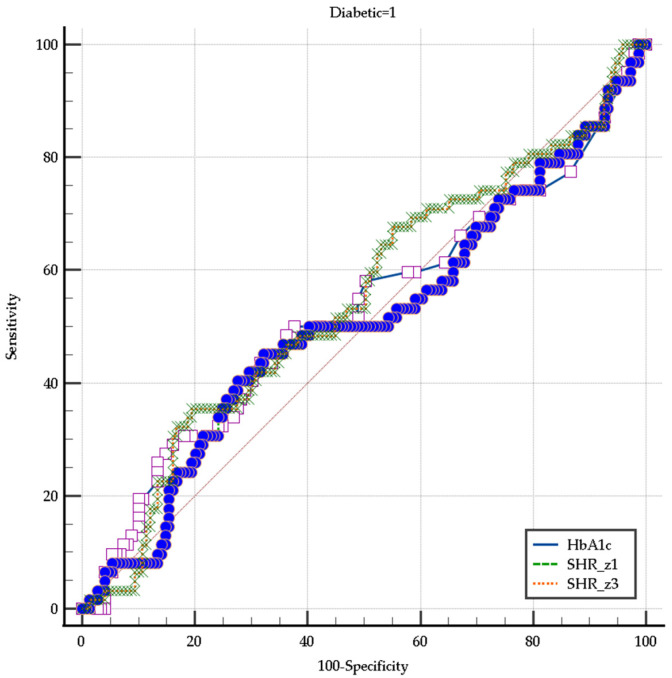
Comparison of Receiver Operating Characteristic (ROC) curves of HbA1c, admission SHR (z1), and 72 h SHR (z3) within the diabetic subgroup (*N* = 211).

While univariable ROC curves identified notable mathematical shifts in thresholds across the cohorts (>1.037 across the total cohort, Figure 4; and >1.329 specifically for diabetic patients; Figure 5), multivariable analysis confirms that the predictive value of these dynamic spikes is accounted for by advanced age and long-standing suboptimal glycemic control (HbA1c). These parameters behave as collinear metabolic features, with chronic disease status heavily driving the dynamic structural failure captured by the SHR at 72 h.

Importantly, while optimal mathematical cut-offs can be extracted using the Youden index for the subgroups (>1.329 for diabetic patients and >0.993 for non-diabetic controls), the underlying univariable ROC curves for these subgroup cohorts failed to reach standalone statistical significance (*p* = 0.3031 and *p* = 0.4431, respectively). These thresholds should be treated as descriptive metabolic characteristics of our specific sample rather than independent, validated clinical targets for triaging. Pair-wise DeLong comparisons across the global cohort indicate that while the chronic glycemic index (HbA1c) maintains clear standalone dominance over both admission and 72 h stress metrics (*p* < 0.05), the absolute global divergence between SHR_z1 and SHR_z3 does not reach independent statistical significance when evaluated univariably (*p* = 0.2224; Table 4 and Table 5). This lack of global separation visually manifests in Figure 4, where the admission and 72 h curves cross repeatedly across mid-range specificities, demonstrating that univariable calculations across mixed populations obscure phenotypic nuances.

**Table 5:** Clinical outcomes and severity.

Outcome Parameter	Non-Diabetic Cohort (*n* = 219)	Diabetic Cohort (*n* = 211)	*p*-Value
Oxygen Requirement			<0.0001
- Grades 0–2 (No/Facial Mask O_2_/CPAP)	211 (96.3%)	168 (79.6%)	
- Grades 3–4 (HFNO/OTI)	8 (3.7%)	43 (20.4%)	
Hospital Discharge Status			<0.0001
- Discharged/Improved	211 (96.3%)	157 (74.4%)	
- Deceased	8 (3.7%)	54 (25.6%)	

Note: Oxygen grade refers to peak support required during hospitalization; because some patients experienced sudden clinical deterioration or terminal events prior to receiving peak non-invasive/invasive ventilation support, mortality numbers may exceed non-fatal Grade 3–4 requirements.

### 3.4. Metabolic Phenotype Stratification

**Figure 6:**
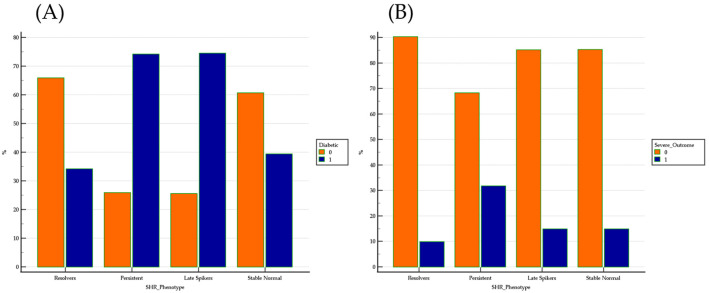
Distribution of SHR phenotypes (Resolvers, Persistent, Late Spikers, and Stable Normal) across (**A**) diabetic status (χ^2^ = 48.94, *p* < 0.0001) and (**B**) severe outcome (χ^2^ = 16.88, *p* = 0.0007).

To translate the continuous SHR_z3 signal into a clinically actionable framework, patients were categorized into four longitudinal metabolic phenotypes based on an SHR cut-off of 1.1 applied at both admission and 72 h: Stable Normal (SHR_z1 < 1.1 and SHR_z3 < 1.1; reference category), Resolvers (SHR_z1 ≥ 1.1 and SHR_z3 < 1.1), Persistent (SHR_z1 ≥ 1.1 and SHR_z3 ≥ 1.1), and Late Spikers (SHR_z1 < 1.1 and SHR_z3 ≥ 1.1) [24]. The distribution of phenotypes across the cohort and the rate of severe outcome within each phenotype are summarized in Table 6 and depicted in Figure 6.

Stratified by diabetic status, the Persistent phenotype was substantially over-represented among diabetic patients (29.9% of diabetic individuals versus 10.0% of non-diabetic controls; χ^2^ = 48.94, df = 3, *p* < 0.0001, contingency coefficient of 0.32), while Stable Normal predominated in the non-diabetic stratum (59.8% of non-diabetic controls). The rate of severe outcome differed significantly across phenotypes (31.8% in Persistent versus 14.8% in Stable Normal, 9.8% in Resolvers, 14.9% in Late Spikers; χ^2^ = 16.88, df = 3, *p* = 0.0007).

A multivariable logistic regression model with SHR_Phenotype as a four-level categorical predictor (Stable Normal as reference) was fitted to evaluate independent phenotype effects after adjustment for age, HbA1c, diabetic status, and viral group (Table 7). The Persistent phenotype carried 81% higher odds of severe outcome compared with Stable Normal (OR = 1.81, 95% CI 0.91–3.60, *p* = 0.0882), with the effect approaching but not reaching conventional statistical significance. Resolvers (OR = 0.65, 95% CI 0.27–1.57, *p* = 0.3347) and Late Spikers (OR = 0.65, 95% CI 0.25–1.69, *p* = 0.3799) showed non-significant trends toward lower odds of severe outcome relative to Stable Normal. Independent predictors retained in this model were age (OR = 1.04 per year, 95% CI 1.02–1.07, *p* = 0.0014) and diabetic status (OR = 3.21, 95% CI 1.34–7.71, *p* = 0.0090), with HbA1c approaching significance (OR = 1.17, 95% CI 0.98–1.39, *p* = 0.0830) once diabetic status was entered as a separate covariate. The phenotype-based model demonstrated the highest apparent (in-sample) discrimination of all models in this analysis (AUC = 0.797, 95% CI 0.756–0.834), with acceptable calibration (Hosmer–Lemeshow χ^2^ = 14.21, df = 8, *p* = 0.0764). Because the four phenotypes were derived post hoc from the same dataset using an SHR cut-off of 1.1, this AUC reflects apparent (optimistic) performance; the value of 0.797 should not be compared directly with the global continuous model without such correction.

**Figure 7:**
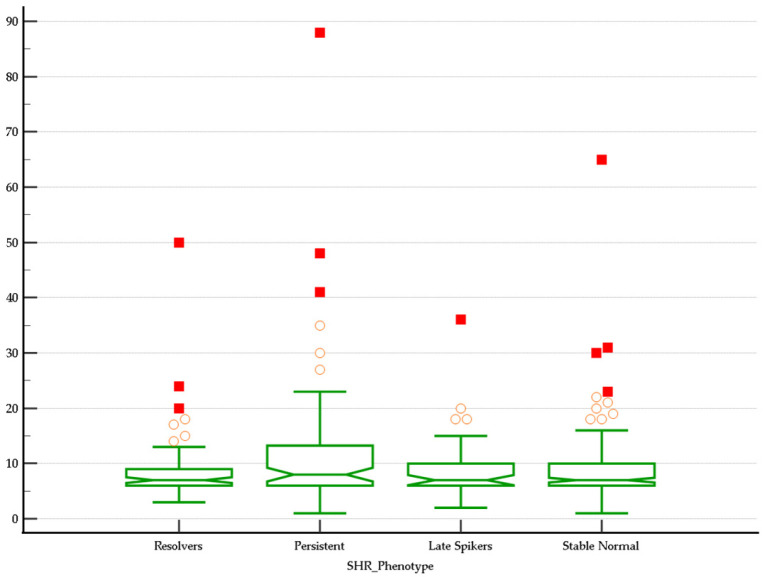
Length of hospital stay (days) across the four SHR phenotypes (Resolvers, Persistent, Late Spikers, and Stable Normal). Kruskal–Wallis H = 13.29 corrected for ties, df = 3, *p* = 0.004. Post hoc Conover comparisons indicate that the Persistent phenotype had significantly longer length of stay than both Resolvers and Stable Normal patients (*p* < 0.05).

Length of hospital stay also differed significantly across the four phenotypes (Kruskal–Wallis H = 13.29 corrected for ties, df = 3, *p* = 0.004). The Persistent phenotype had the highest median length of stay (8 days [IQR 6–13]) and was significantly longer than that of Resolvers (median of 7 days [IQR 6–9]; Conover post hoc *p* < 0.05) and Stable Normal patients (median of 7 days [IQR 6–10]; Conover post hoc *p* < 0.05). Late Spikers (median of 7 days [IQR 6–10]) did not differ significantly from any other group (Figure 7). Although statistically significant, the absolute difference in median length of stay between the Persistent phenotype and the other groups was approximately one day, with broadly overlapping interquartile ranges (Persistent 6–13 days versus Stable Normal 6–10 days); this difference should therefore be regarded as modest in absolute clinical terms and, because length of stay was not adjusted for outcome severity, as hypothesis-generating rather than a direct measure of attributable morbidity.

## 4. Discussion

In the pooled cohort, SHR_z3 was independently associated with severe outcome (OR 1.86, *p* = 0.024). After stratification by diabetes status, this independent association attenuated, although the non-diabetic stratum contained only 12 severe events, so this attenuation likely reflects a combination of true effect modification and limited statistical power.

### 4.1. Clinical Relevance of Longitudinal Metabolic Monitoring

A key finding of this study is the translation of individual metabolic trends into a highly discriminative clinical risk model. As noted during univariate Receiver Operating Characteristic (ROC) analysis, standalone assessment of individual markers like the 72 h stress hyperglycemia ratio offers modest prognostic discrimination when isolated from clinical context (AUC = 0.594), failing to separate cleanly from initial baseline markers under global univariable conditions (*p* = 0.2224). This limitation underscores that metabolic failure does not occur in a vacuum [7].

Our stratified analysis indicates that looking at the 72 h window provides descriptive nuance but lacks independent prognostic power when isolated within distinct metabolic phenotypes [25]. While our global model achieved an acceptable overall Area Under the Curve, clinical stratification revealed that this predictive performance is heavily sustained by underlying static indices rather than acute stress responses. This indicates that persistent stress hyperglycemia does not behave as an isolated, independent driver of pulmonary structural damage but rather functions as a marker that may reflect underlying systemic and neuroendocrine stress governed by the patient’s age and pre-existing metabolic reserves [25,26,27].

Importantly, because HbA1c serves as the denominator in the SHR formula, a substantial portion of the prognostic information carried by SHR_z3 is mathematically embedded in the chronic glycemic baseline. Once HbA1c is entered as a separate covariate within the diabetic stratum, the residual independent signal from SHR_z3 narrows, while HbA1c (OR = 1.21, *p* = 0.040) and age (OR = 1.05, *p* = 0.0036) remain the dominant independent predictors.

The strong association between phenotype membership and diabetic status (contingency coefficient of 0.32, *p* < 0.0001) also contributes mechanistically to the within-stratum attenuation observed: when diabetic status is held constant within a single stratum, the SHR trajectory information overlaps substantially with chronic HbA1c and with the diabetes variable itself, narrowing the residual independent signal attributable to SHR_z3 in isolation [24].

### 4.2. Threshold Variability and Metabolic Resilience

Our univariable exploratory analysis identified distinct descriptive shifts in prognostic thresholds across different patient populations, though these variations must be interpreted with strict statistical caution given that the subgroup-specific ROC curves did not achieve standalone significance (*p* > 0.05). Rather than serving as independent clinical triggers, these thresholds illustrate variations in underlying baseline physiological setpoints:

• Non-Diabetic Controls (>0.993): In individuals without prior glucose impairment, any continuous ratio approaching or exceeding 1.0 (indicating acute stress glucose levels matching or surpassing their estimated chronic baseline) signals an early disruption of homeostatic metabolic control.
• Diabetic Patients (>1.329): In contrast, diabetic patients exhibit a distinct descriptive pattern reflecting chronic metabolic conditioning, where an escalation toward clinical deterioration only manifests within the sample when stress glucose levels exceed their chronic estimated baseline by more than 30% (achieving a descriptive specificity of 80.5%).

This descriptive 30% divergence in thresholds underscores an important pathophysiological concept: our findings suggest that applying a uniform, one-size-fits-all glucose target may not account for underlying metabolic conditioning [8,11]. Instead, acute hyperglycemia must be interpreted relative to the patient’s underlying chronic metabolic background, recognizing that pre-existing disease significantly shifts the window of acute physiological resilience [25,26,28]. We propose that this interpretation is hypothesis-generating biological inference rather than an established finding, as it is derived from subgroup ROC curves that did not reach statistical significance and were based on small event numbers; it therefore requires prospective, adequately powered validation before any physiological or clinical conclusion is drawn.

### 4.3. Clinical Implications

The findings of this study carry several practical considerations for the bedside management of patients hospitalized with viral respiratory infections. First, the measurement of the SHR at admission alone (SHR_z1) appears insufficient to discriminate between patients who will resolve their acute metabolic stress and those who will develop severe outcomes (univariable AUC 0.502; *p* = 0.9615 between outcome groups). Repeating the measurement at 72 h and combining the two time points into a longitudinal phenotype provided substantially better risk stratification (model AUC = 0.797) than any single time point in isolation. Within this framework, the Persistent phenotype, set as SHR ≥ 1.1 sustained from admission to 72 h, identified the highest-risk subgroup, with a severe-outcome rate of 31.8% (versus 14.8% in the Stable Normal group) and a median hospital stay of 8 days (versus 7 days; *p* = 0.004 across phenotypes). This phenotype was enriched among diabetic patients (74.1% of the Persistent group versus 25.9% non-diabetic; *p* < 0.0001), suggesting that the combination of diabetes and failure of metabolic recovery within the first 72 h may flag a particularly vulnerable population worthy of closer observation. Second, because chronic glycemic exposure (HbA1c) and age emerged as the dominant independent predictors within the diabetic stratum (*p* = 0.040 and *p* = 0.004, respectively), our findings reinforce the long-standing principle that pre-admission metabolic optimization remains a foundational element of risk reduction [29]. Finally, the subgroup-specific Youden cut-offs (>0.993 for non-diabetic individuals and >1.329 for diabetic individuals) should be regarded as hypothesis-generating rather than being ready for clinical deployment and require prospective external validation before being incorporated into formal triage pathways.

### 4.4. Strengths

The principal strengths of this study include its prospective design with a pre-specified 72 h landmark; enrolment of a real-world, PCR-confirmed mixed viral respiratory cohort across multiple seasonal surges; use of the Stress Hyperglycemia Ratio to separate acute stress from chronic hyperglycemia; symmetrical multivariable modeling across the total cohort and both metabolic strata; adherence to STROBE reporting; and transparent presentation of non-significant and statistically unstable results rather than selective emphasis on positive findings.

### 4.5. Limitations

Several limitations should be acknowledged when interpreting these findings. First, this was a single-center prospective study conducted at a tertiary infectious diseases and pneumology hospital in Romania, which may limit external generalizability to other healthcare systems with different patient demographics, viral seasonality, or treatment protocols. Second, although the overall sample of 430 patients was adequate, the non-diabetic stratum contained only 12 severe events, yielding an events-per-variable ratio well below conventional thresholds for stable multivariable modeling [30]; the lack of statistical significance for SHR_z3 within this stratum (*p* = 0.6168) therefore likely reflects a combination of limited power and possible true effect modification and cannot be interpreted as definitive evidence of no effect. Third, the SHR ≥ 1.1 cut-off used to define the four longitudinal phenotypes was selected based on the prior literature and convenience and was applied post hoc within the same dataset; the phenotype model and its associated AUC of 0.797 therefore require prospective external validation in an independent cohort before clinical use. Fourth, the SHR formula uses HbA1c-derived estimated average glucose as its denominator and its accuracy may be reduced in patients with anemia, hemoglobinopathies, recent transfusion, advanced renal disease, or other conditions known to bias HbA1c [31]; data on these confounders were not systematically captured. Fifth, multiple comparisons across univariable tests, stratified multivariable models, and pair-wise ROC analyses were not formally adjusted, and individual *p*-values close to the conventional threshold of 0.05 should be interpreted accordingly. Sixth, chronic comorbidities (e.g., hypertension, ischemic heart disease, COPD, and chronic kidney disease) were not systematically recorded or adjusted for; diabetic and non-diabetic patients are likely to have differed in baseline chronic disease burden, so residual confounding by comorbidity cannot be excluded. Finally, although interleukin-6, C-reactive protein, procalcitonin, and D-dimer were measured at admission and on day 6, their longitudinal analysis is beyond the scope of this study and is reported separately; other mediators (e.g., TNF-α and ferritin), insulin resistance indices, and detailed pharmacological exposure profiles were not captured.

## 5. Conclusions

This prospective longitudinal study demonstrates that the dynamic evolution of metabolic stress, rather than absolute admission values, provides important descriptive insights into patient trajectories during hospitalization for acute viral respiratory infections. The clinical stratification reveals that a persistent stress hyperglycemia phenotype at 72 h (SHR_z3) does not function as an isolated, independent driver of severe respiratory decline. While univariable trends identify notable metabolic patterns, fully adjusted multivariable modeling demonstrates that acute 72 h stress metrics are largely secondary to pre-existing chronic metabolic dysfunction and the advanced age of the host.

Our stratified findings lead to several key insights for clinical practice and metabolic research:

• Attenuation of Independent Prognostic Significance: In contrast to global univariable models, stratified multivariable logistic regression adjusted for demographic risk factors and grouped viral etiologies demonstrated that continuous SHR_z3 was not an independent predictor of severity in both the non-diabetic (*p* = 0.6168) and established diabetic (*p* = 0.1954) sub-cohorts. This predictive attenuation indicates that acute 72 h stress fluctuations do not act as standalone clinical targets.
• Primacy of Chronic Metabolic Conditioning and Age: Within the high-risk diabetic population, severe clinical progression is driven independently by chronic background glycemic neglect (HbA1c: *p* = 0.0404, OR = 1.21) and advanced age (*p* = 0.0036, OR = 1.05). Within this cohort, long-standing chronic glycemic and vascular burden appeared to contribute more to clinical risk than the transient stress adjustments captured during early hospitalization, although the study design did not directly measure cellular or vascular conditioning.
• Distinct Predictive Profiles of Glycemic Thresholds: For the total unstratified population, the univariable AUC for SHR_z3 was significantly greater than chance (AUC 0.594, *p* = 0.025), with a Youden-optimal cut-off of >1.037. After clinical stratification, the subgroup-specific ROC curves did not reach statistical significance (*p* = 0.4431 for the non-diabetic threshold of >0.993; *p* = 0.3031 for the diabetic threshold of >1.329). The statistically significant whole-cohort result and the non-significant sub-group thresholds are of different evidential status and must be kept distinct: the subgroup cut-offs should therefore be interpreted as descriptive indicators rather than validated triage triggers.
• Value of Longitudinal Phenotyping: The marked divergence between non-significant admission parameters (SHR_z1) and later longitudinal trends confirms that a 72 h clinical window is necessary to capture a failure of homeostatic metabolic recovery. A persistent stress hyperglycemia phenotype should be viewed as a clinically useful warning signal reflecting an exhausted physiological reserve downstream of advanced age and baseline tissue damage.
• Metabolic Phenotype as Clinical Translation Tool: A longitudinal SHR phenotype model categorizing patients into Stable Normal, Resolvers, Persistent, and Late Spikers achieved the highest in-sample discrimination of all models tested (AUC = 0.797). The Persistent phenotype, defined by SHR ≥ 1.1 sustained from admission to 72 h, showed a trend toward higher adjusted odds (OR 1.81, *p* = 0.088) of severe outcome compared with stable normal patients, consistent with the hypothesis that failure of metabolic recovery within the 72 h window identifies a clinically actionable high-risk group, particularly when combined with diabetic status and advanced age.

Future research is warranted to investigate whether optimizing pre-admission chronic metabolic health (HbA1c) or targeting the downstream pathways of inflammatory aging can successfully stabilize clinical trajectories and improve survival in vulnerable, high-risk patient populations.

## Figures and Tables

**Table 1:** Demographic and clinical characteristics of the study population stratified by diabetic status (*N* = 430).

Variable (Median [IQR])	Non-Diabetic (*n* = 219)	Diabetic (*n* = 211)	*p*-Value
Age (years)	65.0 [45.0–76.0]	73.0 [66.0–79.0]	*p* < 0.0001
BMI (kg/m^2^)	25.4 [22.5–29.1]	29.4 [25.0–34.3]	*p* < 0.0001
Abdominal Circumference (cm)	89.0 [74.0–100.0]	100.0 [88.0–113.8]	*p* < 0.0001
HbA1c (%)	5.2 [5.1–5.4]	7.3 [6.5–9.3]	*p* < 0.0001
SHR (Day 1)	0.99 [0.86–1.19]	1.03 [0.72–1.36]	*p* = 0.8641 (NS)
SHR (Day 3)	0.88 [0.81–1.00]	1.07 [0.76–1.32]	*p* = 0.0002

**Table 2:** Global multivariable logistic regression model for the prediction of severe outcomes in the total study population (*N* = 430). Hosmer–Lemeshow χ^2^ = 13.26, df = 8, *p* = 0.1031. Model AUC = 0.771 (95% CI 0.729–0.810).

Predictor Variable	Coefficient	Standard Error	Wald χ^2^	*p*-Value	Odds Ratio	95% CI
SHR_z3 (72 h)	0.623	0.276	5.093	0.024	1.86	1.09–3.20
HbA1c (Chronic Background)	0.343	0.07	24.332	<0.0001	1.41	1.23–1.62
Age	0.048	0.013	14.64	0.0001	1.05	1.02–1.08
Sex	0.281	0.314	0.798	0.3718	1.32	0.72–2.45
BMI	−0.0002	0.04	0.00001	0.997	1.00	0.92–1.08
Abdominal Circumference (cm)	−0.0012	0.014	0.007	0.935	1.00	0.97–1.03
Viral Group 1 (Influenza/Para)	Reference	Reference	Reference	N/A	1.00	Reference
Viral Group 2 (Coronaviruses)	−0.377	0.33	1.312	0.252	0.69	0.36–1.31
Viral Group 3 (Pneumoviruses)	0.474	0.434	1.193	0.2748	1.61	0.69–3.76
Viral Group 4 (Other Pathogens)	−0.207	0.516	0.16	0.6888	0.81	0.30–2.24

**Table 3:** Stratified multivariable logistic regression models for the prediction of severe clinical outcome (the non-diabetic model contains only 12 severe events [EPV ~ 1.2]; its estimates are statistically unstable and should be read as descriptive/exploratory only).

Predictor Variable	Non-Diabetic Cohort (*n* = 219) P/OR (95% CI)	Diabetic Cohort (*n* = 211) P/OR (95% CI)
SHR_z3 (72 h)	0.6168/1.64 (0.24–11.48)	0.1954/1.51 (0.81–2.83)
HbA1c (Chronic Baseline)	0.2846/2.96 (0.41–21.52)	0.0404/1.21 (1.01–1.46)
Age	0.1665/1.03 (0.99–1.07)	0.0036/1.05 (1.02–1.09)
Sex	0.4801/1.60 (0.44–5.83)	0.6299/1.20 (0.58–2.49)
BMI	0.2404/0.89 (0.74–1.08)	0.5295/1.03 (0.94–1.13)
Abdominal Circumference	0.3721/1.03 (0.97–1.09)	0.4951/0.99 (0.96–1.02)
Viral Group 1 (Influenza/Para)	Reference (1.00)	Reference (1.00)
Viral Group 2 (Coronaviruses)	0.9569/0.96 (0.20–4.57)	0.1910/0.61 (0.29–1.28)
Viral Group 3 (Pneumoviruses)	0.1554/3.87 (0.60–25.04)	0.7407/1.18 (0.45–3.08)
Viral Group 4 (Other Pathogens)	0.2049/3.45 (0.51–23.39)	0.1682/0.43 (0.13–1.43)
Hosmer–Lemeshow Fit	χ^2^ = 5.15, *p* = 0.7410	χ^2^ = 12.39, *p* = 0.1348
Model AUC (95% CI)	0.733 (0.669–0.790)	0.676 (0.608–0.739)

**Table 4:** Pair-wise comparison of Receiver Operating Characteristic (ROC) curves for the prediction of severe outcomes (*N* = 430). Statistical comparison via the DeLong method confirms that while chronic background glycemia (HbA1c) is a significantly stronger global predictor than both stress metrics (*p* < 0.05), the standalone univariable divergence between the admission snapshot (SHR_z1) and the 72 h trajectory (SHR_z3) does not reach independent statistical significance across the unstratified total population (*p* = 0.2224).

Comparison	ΔAUC	SE	95% Confidence Interval (CI)	z-Statistic	*p*-Value
HbA1c vs. SHR_z1	0.228	0.0424	0.145 to 0.312	5.383	<0.0001
HbA1c vs. SHR_z3	0.137	0.0552	0.0283 to 0.245	2.472	0.0134
SHR_z1 vs. SHR_z3	0.0918	0.0752	−0.0557 to 0.239	1.220	0.2224

**Table 6:** Distribution of the four longitudinal SHR phenotypes across the cohort, stratified by diabetic status and severe outcome (*N* = 430). χ^2^ for phenotype × diabetic status: 48.94, *p* < 0.0001. χ^2^ for phenotype × severe outcome: 16.88, *p* = 0.0007.

Phenotype	*n* (% of Cohort)	Non-Diabetic *n* (%)	Diabetic *n* (%)	Severe Outcome *n* (%)
Resolvers	82 (19.1%)	54 (65.9%)	28 (34.1%)	8 (9.8%)
Persistent	85 (19.8%)	22 (25.9%)	63 (74.1%)	27 (31.8%)
Late Spikers	47 (10.9%)	12 (25.5%)	35 (74.5%)	7 (14.9%)
Stable Normal	216 (50.2%)	131 (60.6%)	85 (39.4%)	32 (14.8%)
Total	430 (100%)	219 (50.9%)	211 (49.1%)	74 (17.2%)

**Table 7:** Multivariable logistic regression model for severe outcome with SHR_Phenotype as a four-level categorical predictor (Stable Normal as reference category) (*N* = 430). Model χ^2^ = 70.37, df = 9, *p* < 0.0001; Hosmer–Lemeshow χ^2^ = 14.21, df = 8, *p* = 0.0764; AUC = 0.797 (95% CI 0.756–0.834).

Predictor	Coefficient	SE	Wald χ^2^	*p*-Value	OR (95% CI)
SHR_Phenotype = Resolvers	−0.437	0.453	0.93	0.3347	0.65 (0.27–1.57)
SHR_Phenotype = Persistent	0.596	0.349	2.91	0.0882	1.81 (0.91–3.60)
SHR_Phenotype = Late Spikers	−0.428	0.487	0.77	0.3799	0.65 (0.25–1.69)
SHR_Phenotype = Stable Normal	Reference	—	—	—	1.00 (Reference)
Age	0.042	0.013	10.14	0.0014	1.04 (1.02–1.07)
HbA1c	0.153	0.088	3.01	0.083	1.17 (0.98–1.39)
Diabetic = 1	1.167	0.447	6.83	0.009	3.21 (1.34–7.71)
Viral Group 2 (Coronaviruses)	−0.346	0.325	1.13	0.2883	0.71 (0.37–1.34)
Viral Group 3 (Pneumoviruses)	0.459	0.441	1.09	0.2974	1.58 (0.67–3.75)
Viral Group 4 (Other Pathogens)	−0.445	0.52	0.73	0.3919	0.64 (0.23–1.78)

## Data Availability

Data availability is subject to hospital approval (due to internal regulations of the hospital—Regulation UE No. 679 from 2016 regarding protection of personal data).

## Abbreviations

AUC	Area Under the Curve
BMI	Body Mass Index
CI	Confidence Interval
COPD	Chronic Obstructive Pulmonary Disease
CPAP	Continuous Positive Airway Pressure
CRP-C	Reactive Protein
DM	Diabetes Mellitus
eAG	Estimated Average Glucose
HbA1c	Glycated Hemoglobin
HFNO	High-Flow Nasal Oxygen
hMPV	Human Metapneumovirus
ICU	Intensive Care Unit
IL-6	Interleukin-6
IQR	Interquartile Range
NS	Not Significant
OR	Odds Ratio
OTI	Orotracheal Intubation
PCR	Polymerase Chain Reaction
ROC	Receiver Operating Characteristic
RSV	Respiratory Syncytial Virus
SARS-CoV-2	Severe Acute Respiratory Syndrome Coronavirus 2
SH	Stress Hyperglycemia
SHR	Stress Hyperglycemia Ratio
SHR_z1	Stress Hyperglycemia Ratio at admission (Day 1)
SHR_z3	Stress Hyperglycemia Ratio at 72 h (Day 3)
STROBE	Strengthening the Reporting of Observational Studies in Epidemiology
TNF-α	Tumor Necrosis Factor-alpha

## References

[1] Pyeritz R.E. (2025). Diabetes Mellitus. Emery and Rimoin’s Principles and Practice of Medical Genetics and Genomics.

[2] American Diabetes Association Professional Practice Committee for Diabetes (2026). 2. Diagnosis and Classification of Diabetes: Standards of Care in Diabetes—2026. Diabetes Care.

[3] Lu J.Y., Wilson J., Hou W., Fleysher R., Herold B.C., Herold K.C., Duong T.Q. (2023). Incidence of New-Onset in-Hospital and Persistent Diabetes in COVID-19 Patients: Comparison with Influenza. eBioMedicine.

[4] Thomas S., Ouhtit A., Al Khatib H.A., Eid A.H., Mathew S., Nasrallah G.K., Emara M.M., Al Maslamani M.A., Yassine H.M. (2022). Burden and Disease Pathogenesis of Influenza and Other Respiratory Viruses in Diabetic Patients. J. Infect. Public Health.

[5] Mantovani A., Byrne C.D., Zheng M.-H., Targher G. (2020). Diabetes as a Risk Factor for Greater COVID-19 Severity and In-Hospital Death: A Meta-Analysis of Observational Studies. Nutr. Metab. Cardiovasc. Dis..

[6] Hulme K.D., Gallo L.A., Short K.R. (2017). Influenza Virus and Glycemic Variability in Diabetes: A Killer Combination?. Front. Microbiol..

[7] Perakakis N., Harb H., Hale B.G., Varga Z., Steenblock C., Kanczkowski W., Alexaki V.I., Ludwig B., Mirtschink P., Solimena M. (2023). Mechanisms and Clinical Relevance of the Bidirectional Relationship of Viral Infections with Metabolic Diseases. Lancet Diabetes Endocrinol..

[8] Zhang C., Shen H.-C., Liang W.-R., Ning M., Wang Z.-X., Chen Y., Su W., Guo T.-T., Hu K., Liu Y.-W. (2023). Relationship Between Stress Hyperglycemia Ratio and Allcause Mortality in Critically Ill Patients: Results from the MIMIC-IV Database. Front. Endocrinol..

[9] Ali Abdelhamid Y., Kar P., Finnis M.E., Phillips L.K., Plummer M.P., Shaw J.E., Horowitz M., Deane A.M. (2016). Stress Hyperglycaemia in Critically Ill Patients and the Subsequent Risk of Diabetes: A Systematic Review and Meta-Analysis. Crit. Care.

[10] Roberts G.W., Quinn S.J., Valentine N., Alhawassi T., O’Dea H., Stranks S.N., Burt M.G., Doogue M.P. (2015). Relative Hyperglycemia, a Marker of Critical Illness: Introducing the Stress Hyperglycemia Ratio. J. Clin. Endocrinol. Metab..

[11] Xie H., Hao T., Qi R., Zhang L., An Y., Jia D., Wang H., Niu W., Han X., Sha Y. (2026). Association between Stress Hyperglycemia Ratio and All-Cause Mortality among ICU Patients with Sepsis: A Systematic Review and Meta-Analysis. Front. Med..

[12] Li R., Shen M., Yang Q., Fairley C.K., Chai Z., McIntyre R., Ong J.J., Liu H., Lu P., Hu W. (2023). Global Diabetes Prevalence in COVID-19 Patients and Contribution to COVID-19– Related Severity and Mortality: A Systematic Review and Meta-Analysis. Diabetes Care.

[13] Singh A.K., Khunti K. (2020). Assessment of Risk, Severity, Mortality, Glycemic Control and Antidiabetic Agents in Patients with Diabetes and COVID-19: A Narrative Review. Diabetes Res. Clin. Pract..

[14] Gerganova A., Assyov Y., Kamenov Z. (2022). Stress Hyperglycemia, Diabetes Mellitus and COVID-19 Infection: Risk Factors, Clinical Outcomes and Post-Discharge Implications. Front. Clin. Diabetes Healthc..

[15] Magliano D.J., Boyko E.J. (2025). IDF Diabetes Atlas 11th Edition Scientific Committee. IDF Diabetes Atlas.

[16] USchuetz ANJenkins SGCalfee R., Walsh TJWells D.P., Hollenberg M.T., Glesby J.P. (2016). Impact of Early Detection of Respiratory Viruses by Multiplex PCR Assay on Clinical Outcomes in Adult Patients. J. Clin. Microbiol..

[17] Sberna G., Paba P., Mija C., Costanza G., Brillo F., Grelli S., Maggi F., Lalle E. (2026). Assessment of Multiplex Molecular Tests for Detecting Viral Infections in Lower Respiratory Tract Specimens. J. Mol. Diagn..

[18] von Elm E., Altman D., Egger M., Pocock S.J., Gøtzsche P.C., Vandenbroucke J.P., Initiative S. (2007). The Strengthening the Reporting of Observational Studies in Epidemiology (STROBE) Statement: Guidelines for Reporting Observational Studies. Lancet.

[19] (1997). The Expert Committee on the Diagnosis and Classification of Diabetes Mellitus. Report of the Expert Committee on the Diagnosis and Classification of Diabetes Mellitus. Diabetes Care.

[20] Rui Y., Wu B., Huang C., Li Q. (2025). Association between the Stress Hyperglycemia Ratio and All-Cause Mortality in Critically Ill Patients with T2DM: A Retrospective Study. Front. Endocrinol..

[21] Aon M., Alsaeedi A., Alzafiri A., Al-Shammari A., Taha S., Al-Shammari O., Tawakul M., Alshammari J., Alherz N., Alenezi M. (2022). Stress Hyperglycemia Ratio as a Prognostic Marker in Diabetic Patients Hospitalized with COVID-19. Infect. Dis. Rep..

[22] (2026). MedCalc^®^ Statistical Software.

[23] Wildenbeest J.G., Lowe D.M., Standing J.F., Butler C.C. (2024). Respiratory Syncytial Virus Infections in Adults: A narrative Review. Lancet Respir. Med..

[24] Zheng R., Ni W., Shi Y., Qian S., Lin L. (2026). Persistent Stress Hyperglycemia Trajectories Predict Mortality in Sepsis: A Machine Learning-Enhanced Cohort Study. BMC Infect. Dis..

[25] Forbester J.L., Humphreys I.R. (2021). Genetic Influences on Viral-Induced Cytokine Responses in the Lung. Mucosal Immunol..

[26] Mohd S., Kumar L.L., Harish V., Kumar R., Chaudhary A., Sharma V. (2025). Diabetes Mellitus: Complications, Emerging Therapeutic Targets, and Evolving Treatment Approaches. Obes. Med..

[27] Krinsley J.S. (2008). Glycemic Variability: A strong Independent Predictor of Mortality in Critically Ill Patients. Crit. Care Med..

[28] Marshall R.J., Armart P., Hulme K.D., Chew K.Y., Brown A.C., Hansbro P.M., Bloxham C.J., Flint M., Ronacher K., Bielefeldt-Ohmann H. (2020). Glycemic Variability in Diabetes Increases the Severity of Influenza. mBio.

[29] Cariou B., Hadjadj S., Wargny M., Pichelin M., Al-Salameh A., Allix I., Amadou C., Arnault G., Baudoux F., Bauduceau B. (2020). Phenotypic Characteristics and Prognosis of Inpatients with COVID-19 and Diabetes: The CORONADO study. Diabetologia.

[30] Peduzzi P., Concato J., Kemper E., Holford T.R., Feinstein A.R. (1996). A Simulation Study of the Number of Events per Variable in Logistic Regression Analysis. J. Clin. Epidemiol..

[31] Sacks D.B., Arnold M., Bakris G.L., Bruns D.E., Horvath A.R., Lernmark Å., Metzger B.E., Nathan D.M., Kirkman M.S. (2023). Guidelines and Recommendations for Laboratory Analysis in the Diagnosis and Management of Diabetes Mellitus. Diabetes Care.

